# A Decision Support Tool for Allogeneic Hematopoietic Stem Cell Transplantation for Children With Sickle Cell Disease: Acceptability and Usability Study

**DOI:** 10.2196/30093

**Published:** 2021-10-28

**Authors:** Anirudh Veludhandi, Diana Ross, Cynthia B Sinha, Courtney McCracken, Nitya Bakshi, Lakshmanan Krishnamurti

**Affiliations:** 1 School of Medicine Emory University Atlanta, GA United States; 2 Center for Research and Evaluation Kaiser Permanente Atlanta, GA United States

**Keywords:** decision support tool, sickle cell disease, mobile application, mHealth, pediatrics, transplant, mobile phone

## Abstract

**Background:**

Individuals living with sickle cell disease (SCD) may benefit from a variety of disease-modifying therapies, including hydroxyurea, voxelotor, crizanlizumab, L-glutamine, and chronic blood transfusions. However, allogeneic hematopoietic stem cell transplantation (HCT) remains the only nonexperimental treatment with curative intent. As HCT outcomes can be influenced by the complex interaction of several risk factors, HCT can be a difficult decision for health care providers to make for their patients with SCD.

**Objective:**

The aim of this study is to determine the acceptability and usability of a prototype decision support tool for health care providers in decision-making about HCT for SCD, together with patients and their families.

**Methods:**

On the basis of published transplant registry data, we developed the Sickle Options Decision Support Tool for Children, which provides health care providers with personalized transplant survival and risk estimates for their patients to help them make informed decisions regarding their patients’ management of SCD. To evaluate the tool for its acceptability and usability, we conducted beta tests of the tool and surveys with physicians using the Ottawa Decision Support Framework and mobile health app usability questionnaire, respectively.

**Results:**

According to the mobile health app usability questionnaire survey findings, the overall usability of the tool was high (mean 6.15, SD 0.79; range 4.2-7). According to the Ottawa Decision Support Framework survey findings, acceptability of the presentation of information on the decision support tool was also high (mean 2.94, SD 0.63; range 2-4), but the acceptability regarding the amount of information was mixed (mean 2.59, SD 0.5; range 2-3). Most participants expressed that they would use the tool in their own patient consults (13/15, 87%) and suggested that the tool would ease the decision-making process regarding HCT (8/9, 89%). The 4 major emergent themes from the qualitative analysis of participant beta tests include user interface, data content, usefulness during a patient consult, and potential for a patient-focused decision aid. Most participants supported the idea of a patient-focused decision aid but recommended that it should include more background on HCT and a simplification of medical terminology.

**Conclusions:**

We report the development, acceptability, and usability of a prototype decision support tool app to provide individualized risk and survival estimates to patients interested in HCT in a patient consultation setting. We propose to finalize the tool by validating predictive analytics using a large data set of patients with SCD who have undergone HCT. Such a tool may be useful in promoting physician-patient collaboration in making shared decisions regarding HCT for SCD. Further incorporation of patient-specific measures, including the HCT comorbidity index and the quality of life after transplant, may improve the applicability of the decision support tool in a health care setting.

## Introduction

### Background

Sickle cell disease (SCD) is a chronic blood disorder affecting approximately 100,000 adults and children in the United States [1]. It is characterized by the inheritance of a point mutation in the β-globin gene, leading to the sickle shape of red blood cells. Complications of SCD include painful vaso-occlusive episodes, acute chest syndrome, stroke, splenic sequestration, progressive organ dysfunction, and premature mortality [2-5]. Disease-modifying therapies, such as hydroxyurea, L-glutamine [6], voxelotor [7], and crizanlizumab [8], offer the possibility of long-term amelioration of the disorder. Despite undergoing these therapies, patients with SCD have a diminished quality of life (QoL) and life expectancy, which may be 20 years less than that of the general African-American population in which SCD is most prevalent [4].

Autologous gene therapy (GT) is an emerging treatment based on genetic modification of the patient’s own hematopoietic stem cells to minimize the polymerization of abnormal hemoglobin. Early phase clinical trials of GT suggest that it has the potential to result in long-term amelioration of the disease [9,10]. However, for now, allogeneic hematopoietic stem cell transplantation (HCT) remains the only nonexperimental treatment with curative intent. Although HCT offers the promise of long-term disease amelioration without maintenance medications, it is associated with substantial risks of morbidity and mortality in the short term as well as the risk of new chronic and disabling complications. The safety and efficacy of HCT have improved with advances in supportive care [11]. Clinical trials and registry-based data collected by the Center for International Blood and Marrow Transplant Research, Eurocord, and the European Society for Blood and Marrow Transplantation registries have provided estimates on the survival and risk of HCT-related morbidities for patients with SCD, such as graft failure and graft-versus-host disease (GVHD) which occurs when donor stem cells perceive the recipient’s body as foreign [11-15]. These studies suggest an overall survival (OS) rate for the sampled population of >90%, with the best patient outcomes observed in younger patients with an HLA antigen–identical related donor [11,14,15]. Thus, certain patients with severe manifestations of SCD may benefit from undergoing HCT.

The unavailability of HLA antigen–identical sibling donors and the disparity between physicians' and patients’ or parents’ assessment of the patient’s disease severity may be a barrier to HCT [16-18]. There is an increasing application of HCT for SCD with the advent of HCT from HLA antigen–matched unrelated donors, HLA antigen–haploidentical family donors, and emerging GT [15-19]. The availability of novel treatments, as well as the increased willingness of physicians and patients to consider HCT, may contribute to the increasing uptake of this treatment [20-22]. Previous studies have investigated the decision-making process and preferences of patients and families considering HCT [18,23-27].

### Objectives

Although the reported HCT survival outcomes for patients with SCD are generally favorable, it may be difficult for health care providers to personalize risk factors for an individual patient or have expert knowledge of the field. In addition, although age and type of donor are the most important predictive factors of HCT outcomes, there is no available tool to individualize the risk factors for age, type of donor, type of conditioning, or other factors to provide estimates for an individual patient [11-15]. A decision support tool that incorporates published registry patient data could be used by health care providers to determine personalized risk and survival estimates post-HCT, including overall and event-free survival (EFS) and risk of GVHD, and present them to the patient and their family. The tool may help patients weigh the risks and pros and cons in the context of their own values and preferences. We are not aware of any study or decision support tool that presents personalized transplant outcomes of overall and EFS and risk of GVHD to health care providers. The objectives of this study are to: (1) create a prototype decision support tool that presents personalized risk and survival estimates related to HCT, and (2) determine the acceptability and utility of such a tool for health care providers in helping their patients with SCD make informed decisions regarding HCT*.*

## Methods

### Selection of Registry Data for Predictive Models

In preparation of creating prediction models for HCT outcomes, we analyzed registry-based studies that depicted transplant registry data reported to the Center for International Blood and Marrow Transplant Research, Eurocord, and European Society for Blood and Marrow Transplantation databases [11,14,15]. Studies were selected based on several criteria, including a large patient sample (>100 per donor type) and diversity of patients with respect to age, sex, type of donor, stem cell source, and type of conditioning regimen used for transplant. Three studies were selected to represent the 4 donor types associated with HCT: HLA antigen–matched sibling, HLA antigen–matched unrelated, haploidentical, and HLA antigen–mismatched unrelated. Patient profiles for each donor type are shown in Table 1.

**Table 1:** Selected patient samples from registry data.

Study and stem cell source					Patients (adults and children), n (%)
**Cappelli et al [14]**					
	**HLA antigen–matched sibling**				
		**Age (years)**			
			0-5	175 (23.8)	
			6-15	436 (59.2)	
			>15	125 (17)	
**Gluckman et al [11]**					
	HLA antigen–matched sibling				1000^a^ (100)
**Eapen et al [15]**					
	HLA antigen–matched unrelated				111 (12.2)
	Haploidentical				137 (15.1)
	HLA antigen–mismatched unrelated				104 (11.4)

^a^Adults: 154; children: 846.

All 5 cohorts excluded patients who received uncommon conditioning regimens and inadequate follow-ups after transplant. All 5 cohorts used multivariable survival analysis to estimate hazard ratios for the events of interest. As we did not have access to the raw registry data, the published hazard ratios were extrapolated to estimate survival probabilities for combinations of patient factors.

### Transplant Characteristic Variables

The registry-based studies containing data for the 5 cohorts were examined for OS, EFS, graft failure, and GVHD (acute and chronic). EFS was defined as the percentage of patients who both survived the transplant and did not experience graft failure during the follow-up period. For the purpose of the decision support tool, we only examined demographic information, including sex and age at HCT. Age was regarded as a continuous variable on transplant outcomes, and all possible ages in the analyzed cohorts were included. Transplant characteristics were also examined, including the donor type, stem cell source (bone marrow, peripheral blood, and cord blood), and conditioning regimen (myeloablative, reduced intensity, and nonmyeloablative). Race, ethnicity, and pretransplantation comorbidities such as vaso-occlusion and other chronic organ complications were not regarded for cohort patients.

### Analysis of Registry Data

We conducted an initial analysis of the selected registry data by creating custom data sets in Microsoft Excel. These data sets included the number of patients for each donor type sorted by stem cell source, conditioning regimen, and sex. By assuming a uniform distribution of the data, we were able to divide these data sets into 2 or 3 subsets based on the median age of patients in each cohort.

Next, multivariate survival analysis was performed by examining the hazard ratios for each risk factor of HCT. Hazard ratios were interpreted as relative risks between subpopulations and held constant over the follow-up time after HCT (25-48 months depending on the stem cell source). We calculated the relative risk of transplant options by conducting a proportional analysis. This involved taking the age-based subset directly from the registry data, and for each transplant factor, we multiplied the percentage of patients who shared a variant by the corresponding hazard ratio. This allowed us to determine the relative risks for 2 or more variants for a transplant factor. This process was repeated until we had determined the relative risks for all combinations of transplant factors for each HCT outcome: overall and EFS, GVHD, and graft failure. We repeated this process for all age-based subsets of each of the 4 stem cell sources.

The hazard ratios used were pulled directly from registry-based studies that contained the respective cohorts. This is with the exception of the HLA antigen–matched sibling data, which combined the cohort data of Cappelli [14] with the hazard ratios of Gluckman [11] as the cohort contained more age-based subsets.

### Creation of Transplant Prediction Models

We compiled all the proportional analyses that were conducted on the selected registry data and sorted them by transplant characteristics (sex, donor type, stem cell source, and conditioning regimen). This was done for all 5 HCT outcomes, including OS, EFS, graft failure, acute GVHD, and chronic GVHD. For each patient combination of transplant characteristics, we included proportional analyses for 2 or 3 age-dependent subgroups from our initial analysis. This allowed us to perform statistical regression on our data. From the several types of statistical regressions we analyzed, an exponential regression provided the highest coefficient of determination; therefore, this type of regression was performed for all possible patient combinations of transplant characteristics. We then tested each exponential regression equation with patient ages based on the original cohort with regard to donor type. Although the transplant outcomes of children matched the registry data, transplant outcomes for older adults, in many cases, were poorer than expected; hence, we limited the older ages from being selected on the decision support tool.

### Development of the Decision Support Tool

**Figure 1:**
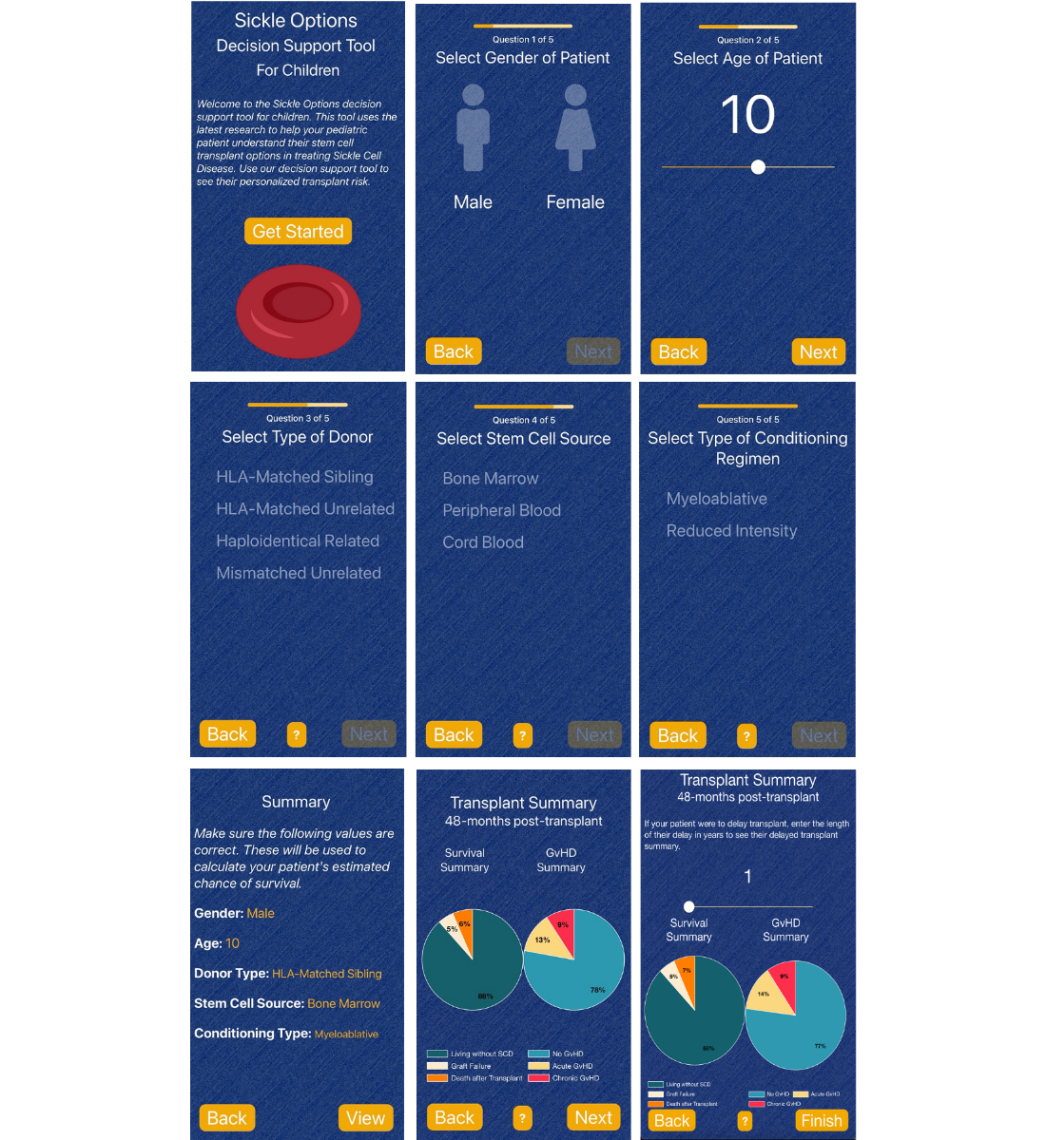
Screenshots of Sickle Options, a decision support tool for children (mobile web app).

The Sickle Options Decision Support Tool for Children is a mobile-focused app that calculates survival and risk estimates for pediatric patients undergoing HCT. The tool was created to help health care providers make more informed decisions regarding their patients’ management of SCD. The decision support tool is coded in HTML and JavaScript using the Monaca Onsen UI 2 framework to allow for a more native mobile user experience. Open-source JavaScript libraries such as Chart.js, which is licensed under the MIT license were incorporated to provide rich graphics for to visual aids. The app was hosted on the Heroku cloud app platform. The support tool provides a brief questionnaire that asks the user 5 basic questions regarding the characteristics of their patients. From this information, the decision support tool is able to display the survival and risk predictions of the selected patient in the form of pie charts and percentages. Help dialogs and tooltips are present to provide context to major keywords and percentages (refer to Figure 1 for screenshots of the Sickle Options Decision Support Tool for Children). Figure 1 depicts individual app screens of entering information for a patient’s transplant to viewing a personalized transplant summary using the prediction model, which contains estimates for OS, EFS, and risk of GVHD.

### Study Design

Following the development of the Sickle Options Decision Support Tool for Children, we conducted alpha testing with a number of staff members familiar with SCD and HCT and made iterative changes in the tool. Once the design of the tool was finalized, we started enrolling participants in qualitative interviews for beta testing and surveys of acceptability and usability. Participants were asked to take part in a 20-minute phone call, starting with a brief overview of the study procedures. After providing verbal consent, participants were then provided with a link to the decision support tool to beta test on their own smartphones. They were asked to express their thoughts and feelings out loud as they proceeded with the tool. Participants were asked to select a transplant for a hypothetical patient before being provided with a personalized survival summary for the patient within the app. Specific questions were posed to the participants based on the particular app screen they were on at the time. The same questions were asked of each participant to maintain consistency. Following their initial run-through of the tool, the participants were asked additional questions pertaining to the tool and for any additional feedback before concluding the interview. After the beta test, participants were asked to complete 2 short surveys, sent via email, on acceptability and usability on REDCap (Research Electronic Data Capture). The interviews were recorded and then transcribed verbatim. Interview transcripts were analyzed using content analysis in Microsoft Excel [28]. The analysis included (1) generating codes based on participant responses to existing interview questions, (2) assessing the insight or other feedback raised by participants in the context of improving the decision support tool, and (3) comparing codes to create common themes exhibited from the data. Participant responses were divided into 4 categories related to user interface, data content, usefulness during patient consultation, and potential for a patient-focused decision aid. A second coder (CBS) reviewed the participant responses and the associated categories to ensure intercoder reliability. Emory University institutional review board provided ethical review and approval for the study (Reference ID: STUDY00000842).

### Recruitment

Heal-Sickle ECHO (Extension for Community Health Care Outcomes) is a telementoring program that uses Project ECHO. Physicians and other health care providers signed up to participate in a 24-week series of fortnightly meetings with didactic presentations and case presentations related to HCT for SCD. Most participants were hematologists, HCT physician faculty, or fellowship trainees. We sent a recruitment email to approximately 65 participants in the Heal-Sickle ECHO series, inviting them to participate in a telephone interview and evaluation of Sickle Options Decision Support Tool for Children for acceptability and usability. Potential users from Heal-Sickle were included if they were (1) physicians who had experience and expertise in providing care to patients with SCD, (2) able to beta test the decision support tool on their phone, (3) willing to complete 2 surveys on acceptability and usability, and (4) willing to participate in a follow-up interview. Although the tool currently provides estimates of HCT outcomes for pediatric patients only, we intend to include estimates for adult patients once more data become available. For this reason, we recruited both pediatric and adult care providers for this study. In total, 18 physicians consented to participate in the study, indicating a response rate of 28%. The participants were compensated with a US $25 gift card for their involvement in the study.

### Qualitative Interview

Study participants were asked for their feedback on the tool through a semistructured qualitative interview. The selection of questions posed to the participants is described in Textbox 1. In addition to these questions, participants were asked to give their thoughts on each screen of the decision support tool and suggest any improvements or modifications to the tool.

**Selections from the qualitative interviews.**

**Questions**

1. How do you feel about the general user interface?
  How easy is it to navigate through the app?
  How useful are the help dialogs and tooltips?
2. What did you think about how the data was visualized?
  What format would you prefer?
  What other information would you like to see visualized?
3. Is there anything you specifically liked about the tool?
4. Is there anything you would like to change about the tool?
5. Do you see a decision support tool like this fitting into your consultations with patients?
  (*If the participant indicated that the tool was suitable*) How will it assist you in decision-making about bone marrow transplant (BMT)?
  (*If the participant indicated that the tool was unsuitable*) How do you think the tool should be changed to your satisfaction?
  Would this information help to ease the decision-making process?
6. We are also planning to create a patient-focused decision aid version of the tool. Any thoughts?
  What things would you like to see changed in the patient decision aid version?
  How will a patient-focused decision aid affect your consultations with your patients?

### Acceptability and Usability Questionnaires

An acceptability survey was created for the decision support tool by adapting the Ottawa Decision Support Framework (ODSF) [29]. This survey measured the tool comprehensibility, presentation of information, and overall suitability for decision-making. This was done using a mixed-scale questionnaire. The rating of sections (bone marrow transplant [BMT], evidence on BMT, and risk factors) was scored on a 4-point Likert scale ranging from 1 (poor) to 4 (excellent). The remaining questions scored the information presented in the tool on a 3-point Likert scale (more, less, and just right) and dichotomous scales of yes or no and easy or difficult. The acceptability survey was adapted from the original ODSF to include references to the tool and omissions regarding the references of information presented on the tool.

Next, a usability survey was created for the tool by adapting the mobile health app usability questionnaire (MAUQ) [30]. This survey measured the tool ease of use and overall usefulness. This comprised an 18-question survey in which participants responded to each statement on a 7-point Likert scale ranging from 1 (strongly disagree) to 7 (strongly agree). The usability survey was not altered in any way from the original MAUQ.

Question items from both surveys were assessed for mean and SD. In addition, open-ended questions were analyzed qualitatively and viewed in conjunction with the participants’ feedback during the beta test.

## Results

### Participant Characteristics

A total of 18 physicians with a background in SCD participated in this study. Of the 18 participants, 9 (50%) were male, and 9 (50%) were female. Most participants had completed their fellowship training (14/18, 78%) and had up to 15 years of medical experience following fellowship (13/18, 72%).

### Qualitative Interview

#### User Interface and Experience

Participants were first asked to describe their experience in using the decision support tool. All participants stated that the tool was easy to use and straightforward overall. In addition, 39% (7/18) of participants who commented directly on the navigation of the tool reported smooth navigation between pages. In particular, participants pointed out the intuitive user experience:

Good size, easy to select stuff, easy to know what I’ve selected if I want to change. Looks pretty fluid.

Participant 7

Further discussion was focused on the individual components and dialogs of the user interface. Help dialogs were activated at the bottom of the tool screen to present additional information pertaining to the respective screen. Of the 6 participants who used these during the beta test, 3 (50%) mentioned that the dialog boxes were useful, whereas 1 (17%) participant indicated no preference. Participants reviewed the 2 transplant summary screens that contained posttransplant risk and survival estimates. Most participants (17/18, 94%) approved of the pie charts on the transplant summary screens as an efficient way to visualize the survival summary:

I like the fact that you're given the percentages with the pie charts. I think that's the most important thing. It updates very quickly. This is great.

Participant 12

However, of the 15 participants who commented directly on the pie chart percentages, 7 (47%) expressed reservations about having to tap on the pie charts to view them. They preferred that the percentages be displayed automatically over the pie chart and suggested that it would save time and be more intuitive. Some participants also reported visual glitches during the beta test. A few participants (5/18, 28%) mentioned that the chart overlapped with the bottom navigation buttons, making it difficult to view it. Finally, 11% (2/18) of participants proposed the idea of a quick-edit menu on the summary page before the transplant summary screens. They attested that a quick-edit menu would be more usable as it would save time when modifying data for a particular patient. Modifications were made to the tool iteratively by incorporating the feedback and suggestions received.

#### Data Content and Significance

Participants were asked to provide feedback related to the pie charts. Of the 11 participants who commented on the information contained in the pie charts, 9 (82%) liked that the displayed pie chart information pertaining to survival, GVHD, and graft failure. In their clinical experience, they believed that these items were most likely what patients would want to know most about transplant:

I think it gives you an idea of living without SCD. Survival summary, and how things are potentially- like, gives you the risk.

Participant 4

A few participants who dealt primarily with adult patients suggested having larger age ranges when selecting a patient’s age. The second transplant summary screen is analogous to the first except that it allows the user to calculate transplant outcomes after specifying the transplant delay in years. Of the 13 participants who commented directly on the delayed transplant feature of the app, 11 (85%) liked the feature of demonstrating a change in outcome when delaying a patient’s transplant as being potentially clinically useful. However, a few participants suggested increasing or refining the range of years for delaying transplant. These participants maintained that some pediatric patients might want to wait until adulthood to make the decision for themselves, whereas others may have reasons to delay the transplant.

Participants provided feedback on the individual risk and survival estimates. A few participants (4/18, 22%) suggested that the GVHD risk summary should be broken down by severity (grade) to better understand a patient’s prognosis after transplant. In addition, some participants (6/18, 33%) proposed that the tool should include information related to GVHD prophylaxis to better understand a patient’s risk of GVHD. A few physicians stated that definitions of the types of conditioning regimens such as myeloablative are very ambiguous in the published literature. Some also indicated that their individual strategies and institutional outcomes for a specific transplant might be at variance from the registry data.

Some physicians (5/18, 28%) suggested that information on the HCT comorbidity index should be incorporated into the tool to allow for more accurate survival estimates as it would affect the success of the transplant. In addition, a few participants requested QoL assessments in the estimates and in the results screens to provide more context to a patient’s posttransplant survival. Some participants indicated that the lack of information on GVHD prophylaxis, HCT comorbidity index, and QoL might limit the utility of the decision support tool to educate patients regarding HCT for SCD:

Quality of life, pain. I mean I'm trying to think of what my patients are going to want to know and they're going to kind of want to know is my life going to get better. Like for a 16-year-old, survival doesn't mean a whole lot.

Participant 15

#### Usefulness to Support Patient Consultations

Participants were asked about the usability of the decision support tool in their own patient consultations. Of the 15 participants who responded to this question, 13 (87%) expressed enthusiasm about the potential use of the tool. Common reasons included tool convenience, risk and survival estimates, and interactivity with patients:

You know, thinking about having something on hand, so I don’t have to search through the literature or go online, which takes a lot of time [...] something that’s easy and readily accessible. Yeah, this is very handy.

Participant 6

Approximately 11% (2/18) of participants expressed reservations about using the tool during patient consultations because of the unavailability of adult data and the fact that the HCT comorbidity index was not included in the current version of the tool. Others mentioned that they would use the tool in certain situations, such as HCT with matched sibling donors or not enrolling a patient for HCT in a clinical trial. Finally, of the 9 participants who were asked directly if the tool would help ease the decision-making process during the consult, 8 (89%) attested that it would. Participants discussed the tool function as a visual aid and the potential to better capture a patient’s attention:

This is very useful for people - for physicians who are - you know doing consults on families and reviewing their results with them so I think it's a useful tool. I do like it. I think it's very user friendly and very simple which is good because it's easy to explain to the families.

Participant 18

#### Potential for a Patient-Focused Decision Aid

Of the 12 participants who commented on a potential patient decision aid, 9 (75%) indicated that the medical terminology currently used in the tool, including the type of donor and conditioning regimen, would need to be simplified in the patient decision aid version. Participants pointed to concerns about a patient’s background and the potential for patients to take results out of context because of low health literacy:

Pie charts are a good way to accurately represent the risks associated with it. It's very visual [...] saying a bunch of numbers but [...] a lot of information in a short period of time for families that may not have high literacy can be very overwhelming.

Participant 8

The participants later discussed the timeline regarding when the patient decision aid should be used. Of the 6 participants, 3 (50%) expressed that the decision aid should be used before a consultation. Participants suggested that using the decision aid before consultation would result in more physician-patient interactions.

Other participants expressed doubts about the patients’ ability to use a decision aid before consultation. They stated that patients might not know what donor or conditioning regimen they were eligible for until meeting with the transplant physician. They also expressed doubts about the potential of the decision aid to steer patients away from transplant.

### Acceptability and Usability Questionnaires

#### Participant Questionnaire Responses

Analysis of participant feedback from postinterview questionnaires on acceptability and usability of the decision support tool centered on 3 major themes of discussion: (1) user interface and experience, (2) data content and significance, and (3) usefulness to support decision-making. Participant responses to the acceptability and usability questionnaires with itemized ratings are further described in Tables 2 and 3, respectively. The acceptability questionnaire uses a mixed Likert scale and a dichotomous scale, whereas the usability questionnaire uses a 7-point Likert scale with a score of 7 indicating *strongly agree*.

**Table 2:** Responses to acceptability questionnaire.

Statements		Value, mean (SD)
**Please rate each section by circling “poor,” “fair,” “good,” or “excellent” to show^a^:**		
	Bone marrow transplantation	2.94 (0.66)
	Evidence about transplantation	2.94 (0.66)
	Risk factors including age, donor type, stem cell source, and conditioning regimen	3.29 (0.47)
The amount of time the learning took was: 1=too long, 2=too short, and 3=just right		2.81 (0.40)
The amount of information was: 1=too much information, 2=too little information, and 3=just right		2.59 (0.51)
I found the learning: 1=slanted towards taking bone marrow transplantation, 2=slanted against taking bone marrow transplantation, and 3=balanced		2.88 (0.49)
Do you find this decision support tool useful while you are making your decision for your patient about bone marrow transplantation? 1=yes and 2=no		1 (0)
What did you think of the way to calculate risk factors with bone marrow transplantation? Was it: 1=easy to find your patient’s risk level, or 2=difficult		1.06 (0.24)
Do you think we included enough information to help someone with sickle cell disease decide whether or not to start bone marrow transplantation? 1=yes, and 2=no		1.53 (0.51)
What did you like about the decision support tool?		—^b^
What suggestions do you have to improve the decision support tool?		—^b^

^a^For the rating of sections, 1=poor, 2=fair, 3=good, 4=excellent.

^b^Participants provided open-ended responses instead of numerical ratings.

**Table 3:** Responses to usability questionnaire

Statements	Value^a^, mean (SD)
The app was easy to use.	6.71 (0.59)
It was easy for me to learn to use the app.	6.81 (0.54)
The navigation was consistent when moving between screens.	6.53 (0.87)
The interface of the app allowed me to use all the functions (such as entering information, responding to reminders, and viewing information) offered by the app.	6.29 (1.10)
Whenever I made a mistake using the app, I could recover easily and quickly.	6.41 (1.06)
I like the interface of the app.	6.35 (0.86)
The information in the app was well organized, so I could easily find the information I needed.	6.29 (0.92)
The app adequately acknowledged and provided information to let me know the progress of my action.	6.29 (0.92)
I feel comfortable using this app in clinical settings.	6.47 (0.94)
The amount of time involved in using this app has been fitting for me.	6.59 (0.62)
I would use this app again.	6.47 (1.01)
Overall, I am satisfied with this app.	5.94 (1.09)
The app would be useful for my health care practice.	6 (1.12)
This app improved my access to delivering health care services.	6.18 (0.88)
This app helped me manage my patients’ health effectively.	5.76 (1.20)
This app has all the functions and capabilities I expected it to have.	5.53 (1.81)
I could use the app even when the Internet connection was poor or not available.	4.88 (1.93)
This mHealth^b^ app provides an acceptable way to deliver health care services, such as accessing educational materials, tracking my own activities, and performing self-assessment.	5.35 (1.50)

^a^Cumulative mean, 6.15 (SD 0.79).

^b^mHealth: mobile health.

#### User Interface and Experience

As shown in Table 3, the usability of the decision support tool was shown to be high, with overall usability of 6.15 (SD 0.79; range 4.2-7) on the MAUQ. This is reflected in the high scores on statements related to ease of use (mean 6.71, SD 0.58; range 5-7), navigation (mean 6.53, SD 0.89; range 4-7), user interface pertaining to functionality (mean 6.29, SD 1.14; range 4-7) and overall look (mean 6.35, SD 0.87; range 5-7). The participants were also pleased with the length of the tool and the amount of time it took to complete. This statement was scored high on both the acceptability questionnaire (mean 2.81, SD 0.41; range 2-3; Table 2) and usability questionnaire (mean 6.59, SD 0.62; range 5-7).

Participants scored the web-based app low on question 17 of the MAUQ, which asked about using the app during a poor internet connection. This question had a mean score of 4.88 (SD 1.98; range 1-7; Table 3). This is in accordance with statements made by participants during the interview that mentioned spotty Wi-Fi at their medical office. These statements provide support for creating a downloadable app that can be used offline. In addition, a few participants indicated user interface glitches related to visual components in the suggestions portion of the acceptability questionnaire, matching the feedback given during the qualitative interviews.

#### Acceptability Data Content and Significance

In the acceptability questionnaire, participants suggested that the tool was not biased for or against HCT (mean 2.88, SD 0.5; range 1-3) and was easy to calculate risk factors with (mean 1.06 SD 0; range 1-2). Participants also indicated that they were satisfied with the types of information present on the tool, including BMT (mean 2.94, SD 0.63; range 2-4) and risk factors (mean 3.29, SD 0.48; range 3-4; Table 2). With regard to the amount of information presented in the tool, the participants were split. This statement was scored with a mean of 2.59 (SD 0.5; range 2-3), indicating a mix of *too little* and *just right*.

Some participants were not satisfied with the functions and capabilities of the tool, as shown in the usability questionnaire (mean 5.53, SD 1.61; range 2-7; Table 2). In the acceptability questionnaire, participants were asked to provide suggestions for improving the decision support tool. Most suggestions were focused on adding more context to the risk and survival summaries the tool presented. These include adding the time period for registry data, HCT comorbidity index, and type of chemotherapy in relation to the conditioning regimen used. All suggestions given in the acceptability questionnaire matched the feedback given in the qualitative interviews. Participant responses were mixed regarding whether the tool provided enough information as well as its overall acceptability to deliver health care services. In the acceptability questionnaire, participants were split regarding whether the tool provided enough information to help someone with SCD decide on HCT (mean 1.53, SD 0.52; range 1-2; Table 2). This rating is analogous with an earlier statement in the acceptability questionnaire, which asked about the amount of information in the tool.

#### Usability

Starting with the MAUQ in Table 3, participants indicated their approval of the organization of information in the tool (mean 6.29, SD 0.95; range 4-7). As shown in Table 3, participants attested in the MAUQ that the tool would be useful for their health care practice (mean 6, SD 1.02; range 3-7), improve access to delivering health care services (mean 6.18, SD 0.86; range 4-7), and help manage their patients’ health effectively (mean 5.76, SD 1.22; range 3-7). In the acceptability questionnaire, all participants indicated that the decision support tool would be useful when making the decision for their patients about HCT (mean 1, SD 0; Table 2).

In the MAUQ, participants were asked whether the tool provided an acceptable way to deliver health care services, including access to materials, activity tracking, and self-assessment performance. This statement was rated with a mean score of 5.35 (SD 1.54; range 2-7), indicating that participants *somewhat agreed* with the statement (Table 3). These lower ratings are likely representative of the desired additional features that participants requested during qualitative interviews and while answering questionnaires on acceptability and usability. Although some of these features are dependent on research being available in the public domain, such as the HCT comorbidity index affecting a specific type of HCT, others, such as adding the time period for registry data, can be addressed independently.

## Discussion

### Principal Findings

In this study, we report the development, usability, and acceptability of the Sickle Options Decision Support tool for HCT to clinicians in pediatric SCD. These data provide a proof-of-concept of the potential acceptability and utility of such a decision support tool for use by hematologists and HCT physicians. To the best of our knowledge, this is the first decision support tool to provide individualized and age-specific risk and survival estimates for pediatric patients considering HCT. Brazauskas et al [13] published a risk prediction model for patients with SCD based on age and donor type. The model was derived from large registry-based data sets [11,14,15]. These studies report age as a continuous variable for outcomes of HCT for SCD; however, the model does not individualize the risks of outcomes for a given patient. Age was correlated with OS, EFS, and GVHD risk. The lack of individualization of outcomes that families may consider important in decision-making regarding HCT is a barrier to decision-making.

The Sickle Options Decision Support Tool for Children was designed to provide health care providers specializing in SCD with individualized risk and survival estimates for their pediatric patients considering HCT. The tool risk prediction models were made possible by large-scale clinical studies that depicted registry data on transplant outcomes in thousands of patients with SCD [11,14,15]. With this information, the tool can present transplant outcomes based on a patient’s sex, age, donor type, stem cell source, and type of conditioning regimen used in HCT. The decision support tool provides outcomes based on the current age of the patient as well as a selected future age of the patient. Caregivers may exhibit mixed perceptions of HCT for their child in relation to the risk of death or other HCT-related complications (graft failure and GVHD) when making decisions. In addition, although some patients and caregivers may have an interest in wanting to learn more about HCT, there may still be a decisional dilemma, especially when deciding whether to postpone transplant [18]. The use of the decision support tool may help fill a potential information gap by either the patient or the health care provider as well as encourage physician-patient collaboration regarding the decision-making process.

The decision support tool is available as a mobile web app. We conducted a comprehensive beta test of this mobile app and qualitative interviews with 18 hematologist–oncologists with a special interest in curative therapies for SCD. The participants found the decision support tool to be usable and acceptable based on the scoring frameworks of the MAUQ and ODSF, respectively.

Decision support tools can play a useful role in a health care setting in assisting health care providers with the delivery of individualized evidence-based care to their patients. Decision support tools may be used to present a visual display of data to educate patients regarding the risks, benefits, and outcomes of HCT. Thus, they can be used as tools to enhance patient knowledge and engagement, improve standards of care, and help clinicians analyze large sets of data quickly given the time-sensitive nature of decision-making [31]. The development of this tool was largely informed by experience with using the app iChoose Kidney, an app-based decision aid, which uses risk prediction models to help identify whether a patient would be best suited for kidney transplant or dialysis [32]. We believe that a mobile-focused decision support tool will be easily accessible for use by health care providers during their patient consultations and present clear visuals that both health care providers and patients can understand.

Participants engaged in beta testing of the decision support tool provided feedback and made several suggestions that allowed us to refine the decision support tool. We optimized the display of the pie chart by adding automatic percentages to the view and also introduced quick-edit menus to switch between different types of transplants. In addition, after transplant and when it is available, we intend to add information pertaining to QoL in future versions of the decision support tool.

Regarding the comorbidity index reported by registry studies, the index includes factors that do not overlap with the factors found to predict HCT risk and survival outcomes. Eapen et al [15] reported that patients who underwent HCT with a haploidentical donor were much more likely to have an HCT comorbidity index of >3 than patients who underwent HCT with any other type of donor. The lack of impact of the comorbidity index on post-HCT outcomes is likely reflective of the predominantly young patient population in the registry data. However, as more adults with advanced progressions of SCD undergo HCT, it is possible that the HCT comorbidity index may be more of a factor in predicting post-HCT outcomes. We intend to include comorbidity data in future versions of the decision support tool when such a determination is made.

### Limitations

We recognize several limitations of this study. Qualitative content analysis also brought to light the limitations of the current data in providing individualized risk. First, currently available data do not permit the incorporation of patient characteristics, including comorbidities, severity of SCD, organ damage or QoL, chronic pain, or psychological comorbidities into the risk prediction models of the tool. Thus, there are inherent limitations to individualizing risk estimates and predicting outcomes. The availability of such information and its impact on outcomes would provide a more complete picture of a patient’s risk and survival outcomes after undergoing HCT. Currently, there is research based on the effect of the pre-HCT comorbidity index on overall after transplant [33]. On the basis of the outcomes of these studies, we plan to incorporate this information and other information regarding patient comorbidities in future versions of the decision support tool. Second, we developed this prototype app based on published data that contained registry data tables [11,14,15]. Therefore, we make some assumptions regarding the exact number of individuals with a specific donor type, stem cell source, and conditioning regimen for a given age based on cumulative data published. As a result, rounding off may present some error when providing the risk and survival summary for a specific patient, and we only present this tool as a prototype to provide a proof-of-concept for such a tool. To properly assess the tool validity, the next step would be to obtain the raw patient data so that we may construct appropriate multivariate statistical models that incorporate several risk factors of HCT, including the HCT comorbidity index. We are in the process of obtaining the study data set on HCT of 1518 patients with SCD aged <1 to 58 years transplanted in the United States from 106 transplant centers from the Biologic Specimen and Data Repositories Information Coordinating Center, which has been established by the National Heart, Lung, and Blood Institute (NHLBI). The Biologic Specimen and Data Repositories Information Coordinating Center combines the resources of the NHLBI Biologic Specimen Repository, which has been managed by the division of blood disease resources since 1975, and the NHLBI Data Repository, which has been managed by the division of cardiovascular sciences since 2000. We will perform validation studies of the app on this study data set and finalize the development of the app. Third, the registry data we used comprises transplants completed over a wide time frame—1986-2017 for matched sibling donor data [14] and 2008-2017 for all remaining donor data [15]. Gluckman et al [11] reported an era effect in the outcome of HCT for SCD. Refinements in supportive care may render much of the registry data out of date as they may not consider the latest clinical practices in supportive care and the prevention and treatment of GVHD and graft failure. Fourth, the completion of ongoing clinical trials of haploidentical donor BMT in adults and children with SCD or autologous GT may add new dimensions to the consideration of curative options. Fifth, the published registry data may not account for underreporting of outcomes, institutional volume, center experience effect, or physician expertise. Institutions that perform more transplants for SCD may see better outcome success than smaller institutions that perform fewer transplants. Thus, some physicians with experience and expertise in the field expressed hesitation in using the decision tool as they felt that the outcomes at their institutions were likely to be superior to those reported by the registry. This, of course, may also reflect an unconscious bias. These findings suggest the need for future studies examining outcomes with center experience in performing HCT for SCD. Such data will allow physicians to generate individualized estimates of outcomes in centers, such as their center, and they are more likely to use the decision support tool for determining individualized estimates of outcomes and sharing them with their patients.

Some physicians expressed a desire to see QoL pre-HCT and the anticipated impact of HCT on QoL in the risk prediction model of the decision support tool. Studies of decision-making by patients suggest that patients and families consider QoL important in decision-making about HCT [18,23-27]. Recent clinical trials have suggested that HCT can improve QoL after HCT [34-40]. Uniform and systematic collection of QoL before and after HCT in clinical trials and registries is necessary before such data can be incorporated into a decision support tool. Some participants mentioned the desirability of incorporating comparison data on anticipated outcomes with non-HCT disease-modifying therapies. This would, of course, require the availability of such comparative data in large clinical trials.

As most data on HCT for SCD deals with pediatric outcomes, we limited the decision support tool to children. However, future app versions may incorporate adult outcomes because of the rapid increase in the uptake of HCT in adults with SCD and the potential of GT to become the standard treatment.

We recruited participants exclusively from the Heal-Sickle ECHO program for the study. As these physicians have a declared interest in curative therapy and decision-making about HCT for SCD, their perspective may not be generalizable to that of hematologists and transplant physicians who may be less involved in the field. On the other hand, the experience and expertise of the participants were extremely useful in optimizing the design of the tool and identifying future areas of research. Participants in the study also made several recommendations for a proposed version of the decision support tool specifically designed for patients and families. Considering the technical nature of information regarding myeloablation, conditioning regimen, and donor types, the decision support tool may be inherently better suited for use with guidance by physicians who could then use the tool to display individualized risk to their patients. Finally, the response rate of the physicians for the study was low—at 28%. A potential reason for the low response rate may be physician preferences for more traditional modes of research, such as a paper survey rather than a web-based survey [41]. In addition, as the decision support tool was limited to pediatric patients with SCD, some physicians who predominantly treated adult patients with SCD may have considered the app to be beyond the scope of their practice.

### Conclusions

We report the development, beta testing, usability, and acceptability of a decision support tool for individualizing estimates of outcomes of HCT for patients with SCD. Refining the predictive algorithms for era and center experience, incorporating data on QoL, comparison of other disease-modifying therapies, outcomes in adults, and autologous GT offers he possibility of expanding the applicability of such a decision support tool in helping shared decision-making in HCT for SCD.

## Abbreviations

BMT: bone marrow transplant
ECHO: Extension for Community Health Care Outcomes
EFS: event-free survival
GT: gene therapy
GVHD: graft-versus-host disease
HCT: allogeneic hematopoietic stem cell transplantation
MAUQ: mobile health app usability questionnaire
NHLBI: National Heart, Lung, and Blood Institute
ODSF: Ottawa Decision Support Framework
OS: overall survival
QoL: quality of life
REDCap: Research Electronic Data Capture
SCD: sickle cell disease
